# *Rhodiola* pre-conditioning reduces exhaustive exercise-induced myocardial injury of insulin resistant mice

**DOI:** 10.1038/s41598-022-20376-4

**Published:** 2022-11-23

**Authors:** Baiyang You, Jing Cheng, Yaoshan Dun, Jeffrey W. Ripley-Gonzalez, Jie Liu, Dezhao Li, Siqian Fu, Chuangxiong Hong, Suixin Liu

**Affiliations:** 1 The First Clinical Medical College, Guangzhou University of Chinese Medicine, Guangzhou, Guangdong China; 2 Division of Cardiac Rehabilitation, Department of Physical Medicine and Rehabilitation, Xiangya Hospital Central South University, Changsha, Hunan China; 3 Department of Cardiovascular, Shenzhen District Yantian People’s Hospital, Shenzhen, Guangdong China; 4 National Clinical Research Center for Geriatric Disorders, Xiangya Hospital Central South University, Changsha, Hunan China; 5 Division of Preventive Cardiology, Department of Cardiovascular Medicine, Mayo Clinic, Rochester, MN USA; 6 Department of Internal Medicine, School of Medicine, Hunan Traditional Chinese Medical College, Zhuzhou, Hunan China

**Keywords:** Cardiovascular biology, Mitochondria, Drug therapy

## Abstract

Myocardial injury reduction and recovery under acute cardiac stress are adversely impacted by insulin resistance (IR). We previously demonstrated that *Rhodiola* improved cardiac anti-stress capacity in mice. Thus, this study focuses on the preventive efficacy of *Rhodiola* on exhaustive exercise (EE)-induced myocardial injury of IR mice. An 8-week high-fat diet (HFD) model of IR mice was established. *Rhodiola* was administrated by garaging. After the 8-week intervention, half of the mice performed EE to simulate acute cardiac stress, and determine myocardial injury; The remaining mice were sacrificed following fasting to assess metabolic disorder. We found myocardial injury induced by EE in IR mice was worse and was alleviated by *Rhodiola* pre-conditioning. Further, the nuclear factor erythroid 2-related factor 2 (Nrf2)-related antioxidant system was impaired by HFD, while mitochondrial dynamic fusion and fission were activated by HFD as a physiological protective compensation. The *Rhodiola* administration rescued Nrf2 impairment and further facilitated mitochondrial fusion and fission. All these results indicate that *Rhodiola* is a potential treatment for the prevention of cardiac events in type 2 diabetes mellitus and metabolic syndrome patients, and the Nrf2-related antioxidant activity and mitochondrial dynamics are the proposed mechanisms.

## Introduction

Organ adaptability, or anti-stress capacity, is the damage reduction and functional recovery from an acute pathological stimulus. Insulin resistance (IR), as the basis of cardiovascular disease, was found to adversely impact cardiac anti-stress capacity. For IR or diabetic patients, the survival rate and prognosis of acute stress including myocardial infarction, are poor^1,2^, which indicates that cardiac anti-stress capacity is impaired by IR. In our previous research, we found that autophagy, heat shock protein 70 (HSP70), and mitochondrial quality control were found to be vital in anti-fatal stress capacity in healthy mice^3–5^.

Due to the huge energy consumption in the myocardium, myocardial cells contain abundant mitochondria indicating the fundamental role of the mitochondrion in cardiac anti-stress capacity^6^. Moreover, mitochondrion produces an important by-product, reactive oxygen species (ROS), which influences almost all physiological functions, and even damages the mitochondrion in return^7^. The mitochondrial dynamic, which involves the fission and fusion of mitochondrial membranes, is an important component of the quality control system, especially in cardiac cells^8^. Mitochondrial fission participates in the disposal of damaged mitochondria, while fusion is related to the preservation of ATP production and ROS dissipation^9^. It is the Fission and fusion, that determine the structural and functional status of mitochondria^10^.

Evidence has reported that mitochondrial dynamic is associated with ROS production and scavenging^11^. The cellular oxidizing conditions determine mitochondrial fusion and fission by modulating the activity and expression of dynamic proteins, including OPA1 and MFNs^12,13^. Previous studies have shown that defects in mitochondrial dynamic lead to increased ROS, thereafter resulting in reduced ATP production, and exacerbated diabetic nephropathy^14^. All these indicate the correlation between mitochondrial dynamic and ROS is critical in maintaining mitochondrial hemostasis.

In the myocardium of diabetic mice, mitochondrial dynamic disorder, as well as increased oxidative stress has been observed^15^, which could be one of the reasons behind poor cardiac anti-stress capacity in IR patients. However, further research is needed to explore its mechanisms and to develop efficient therapies.

*Rhodiola* (RS), a natural herb historically used to improve body hypoxia resistance at high altitudes^16^, has been shown to be a mitochondrial protectant^17,18^. Our previous study found *Rhodiola* enhances mitochondrial quality including mitochondrial dynamic, to increase cardiac anti-stress capacity in healthy mice^5^. However, whether this improvement can be extended under IR conditions and its underlying mechanism remains unclear. Recently, *Rhodiola* was found to be a Nuclear factor erythroid 2-related factor 2 (Nrf2) activator, which enhances the antioxidant system to exert neuroprotective effects^19^ or relieve hypoxia-induced liver injury^20^. Thus, we hypothesize that *Rhodiola* activates Nrf2 and the downstream antioxidant system to regulate mitochondrial dynamic homeostasis, finally strengthening cardiac anti-stress capacity under IR conditions.

In this study, a high-fat diet (HFD) mice model was established to simulate the vulnerability to cardiac stress in patients with IR, and exhaustive swimming exercise was used to simulate acute cardiac stress to induce myocardial injury. Then, we investigate *Rhodiola* pre-conditioning’s effect on the cardiac anti-stress capacity of HFD mice and explore the role of the Nrf2 signaling pathway.

## Results

### *Rhodiola* reduces exhaustive exercise-induced myocardial mitochondrial injury of HFD Mice

**Figure 1:**
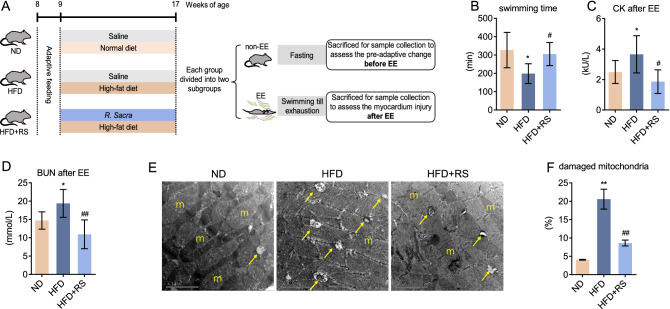
*Rhodiola* reduces exhaustive exercise-induced myocardial injury of HFD Mice. (**A**) After a 1-week adaptive diet, the mice were fed a normal diet (ND) or high-fat diet (HFD) with either *Rhodiola* (RS) for 8 weeks, then mice underwent exhaustive swimming (EE) or not. (**B**) The forced swimming time was recorded and analyzed; (**C**,**D**) Blood urea nitrogen (BUN), and serum creatine kinase (CK) of mice were measured after EE; (**E**) The morphology of myocardium after EE was observed by transmission electron microscope (TEM), intact mitochondria (the letter m), as well as damaged mitochondrion (yellow arrows), were indicated in images, scale bar = 2 μm; (**F**) The ratio of damaged mitochondria number, which have ruptured cristae and edema, to total mitochondrial number per field-of-view was analyzed. Data are expressed as Mean ± SD, n = 6, *, ** represent *P* < 0.05, *P* < 0.01 in comparison with ND; #, ## represent *P* < 0.05, *P* < 0.01 in comparison with HFD.

After adaptive feeding, the mice were fed a normal diet (ND) or HFD with/without *Rhodiola* administration, then the mice underwent exhaustive swimming or not. The animal protocol is further described in Fig. 1A. We found the forced swimming time had decreased in the HFD group compared to ND. However, when accompanied by *Rhodiola* treatment, the mice following HFD demonstrated a comparable swimming time to that of the ND group (Fig. 1B). Following exhaustive swimming, myocardium damage was subsequently evaluated by serum creatine kinase (CK), blood urea nitrogen (BUN), and electron microscopy. We found that serum CK and BUN were increased in HFD mice (Fig. 1C,D). In parallel, damaged mitochondrial with edema and ruptured cristae (yellow arrows) in the myocardium were observed with greater abundance in HFD mice (Fig. 1E,F). However, neither the morphologic alteration in myocardium nor CK and BUN release occurred in HFD + RS groups (Fig. 1C–F), suggesting that *Rhodiola* prevents EE-induced cardiac mitochondrial injury in HFD mice.

### *Rhodiola* relieves the insulin resistance of HFD mice

**Figure 2:**
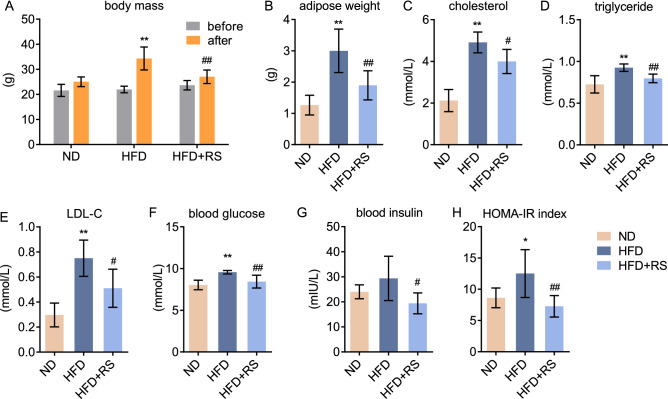
*Rhodiola* relieves the insulin resistance of HFD mice. The mice were fed a normal diet (ND) or a high-fat diet (HFD) with either *Rhodiola* (RS) for 8 weeks, after 5-h fasting they were sacrificed to assess pre-adaptive changes. (**A**) Bodyweight of mice before and after intervention was monitored, (**B**) visceral adipose weight of mice was recorded; (**C**–**G**) Serum cholesterol, triglyceride, LDL-cholesterol, blood glucose, and blood insulin were assessed; (**H**) HOMA-IR index was calculated. Data are expressed as Mean ± SD, n = 6, *, ** represent *P* < 0.05, *P* < 0.01 in comparison with ND; #, ## represent *P* < 0.05, *P* < 0.01 in comparison with HFD.

The HFD mouse model was characterized by dyslipidemia, which indicates lipotoxicity and energy oversupply. An 8-week HFD led to increased weight gain compared to ND mice, while *Rhodiola* administration restrained this tendency in HFD mice even comparable to that in ND (Fig. 2A). In-keeping with the bodyweight result, visceral fat weight in HFD mice also increased, while *Rhodiola* prevented this change (Fig. 2B). As for glucolipid metabolism, we found serum cholesterol, triglyceride, low-density lipoprotein (LDL), blood glucose, and insulin increased in HFD mice, in which the HOMA-IR index also increased. However, *Rhodiola* administration prevented these changes in HFD mice (Fig. 2C–H).

### *Rhodiola* activates Nrf2-related antioxidants in HFD mice

**Figure 3:**
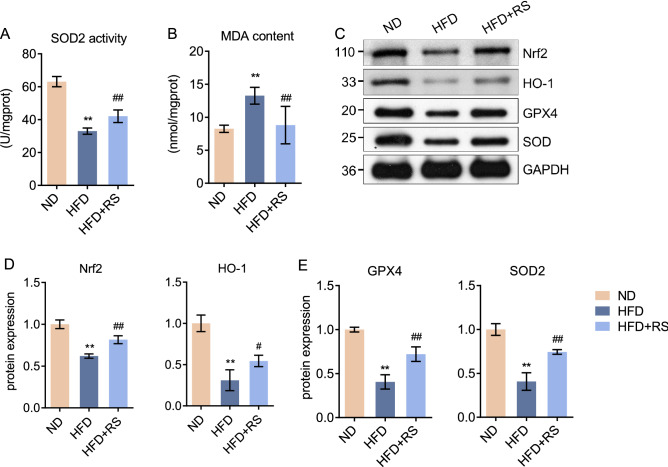
*Rhodiola* activates Nrf2-related antioxidants in high-fat diet mice. After an 8-week intervention and 5-h fasting, the mice were sacrificed to assess the pre-adaptive changes. (**A**,**B**) The myocardium samples were obtained, MnSOD activity and MDA content were assessed; (**C**,**D**) And relative protein expression of Nrf2, HO-1, GPX4, and SOD2 were assessed, and the original blots are presented in Supplementary Figure 3. Data are expressed as Mean ± SD, n = 3, *, ** represent *P* < 0.05, *P* < 0.01 in comparison with ND; #, ## represent *P* < 0.05, *P* < 0.01 in comparison with HFD.

Considering the close correlation between oxidative stress and acute exercise stress in the myocardium, we evaluated oxidative stress and Nrf2-related antioxidant signaling. As shown in Fig. 3A,B, the activity of SOD2 decreased in HFD mice, while the content of malondialdehyde (MDA) increased, indicating oxidative stress. *Rhodiola* administration alleviated HFD-induced oxidative stress. To investigate the role of Nrf2 signaling, we assessed the protein expression of Nrf2 and its downstream HO-1. Our results showed that *Rhodiola* improved inhibited Nrf2/HO-1 pathway in HFD mice (Fig. 3C,D). Protein expression of GPX4 and SOD2 exhibited a similar tendency (Fig. 3E).

### *Rhodiola* prevents mitochondrial dynamics disorder in high-fat diet mice

**Figure 4:**
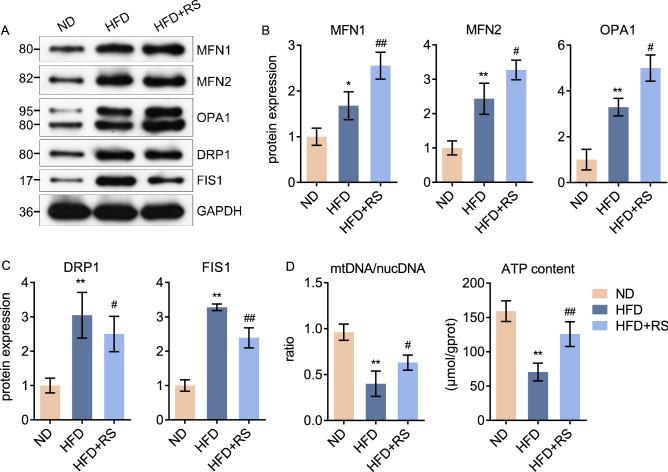
*Rhodiola* prevents mitochondrial dynamics disorder in high-fat diet mice. After an 8-week intervention and 5-h fasting, the mice were sacrificed to assess the pre-adaptive changes. (**A**–**C**) The myocardium samples were obtained, relative protein expression of mitochondrial fusion marker MFN1, MFN2, OPA1 as well as fission marker DRP1 and FIS1 were determined, and the original blots are presented in Supplementary Figure 4; (**D**) The ratio between mitochondrial DNA (mtDNA) and nuclear DNA (nucDNA) determined, and the ATP content of myocardium was detected. Data are expressed as Mean ± SD, n = 3, *, ** represent *P* < 0.05, *P* < 0.01 in comparison with ND; #, ## represent *P* < 0.05, *P* < 0.01 in comparison with HFD.

Mitochondrial dynamic plays a vital role in maintaining the mitochondrial function, which supplies energy for cardiac contractile function. To evaluate the degree of fusion and fission in the myocardium, we assessed fusion factors MFN1, MFN2, and OPA1, as well as fission factors DRP1 and FIS1 expression. As shown in Fig. 4A–C, mitochondrial fusion and fission were both activated in HFD mice. This might be a stress cellular condition due to HFD. However, *Rhodiola* administration changed this: mitochondrial fusion was further enhanced in HFD + RS mice, while fission was prevented. Moreover, the ratio of mitochondrial DNA and ATP content was assessed to evaluate mitochondrial content and function. We found that mitochondrial DNA and ATP content decreased in the myocardium of HFD mice when compared with ND mice, *Rhodiola* treatment reversed this (Fig. 4D).

## Discussion

This study aimed to illustrate the mechanism through which *Rhodiola* improves cardiac anti-stress capacity impaired by HFD in an IR mice model. Our results showed that an HFD increases EE-induced cardiac injury in mice, inhibits Nrf2 signaling regulated antioxidant enzyme, and triggers mitochondrial dynamic disorder. However, all these were alleviated by *Rhodiola* pre-conditioning. These findings suggest that *Rhodiola* is a potential clinical therapy for IR patients to improve cardiac anti-stress capacity.

There are several methods to stimulate cardiac stress, including myocardial infarction, ischemia/reperfusion, chemical drugs, and EE. In comparison with other models, EE would better represent a complicated and changeable condition in clinical settings. Thus, we investigated anti-stress capacity in an EE model. Research by Behringer and colleagues has demonstrated that mitochondrial dysfunction due to EE leads to muscle injury characterized by increased oxidative stress, cytoskeletal damage, and various markers^21^. In addition, it has been demonstrated that EE regulates the Bcl-2 family via PI3K/AKT signaling pathway to decrease the openness level of mPTP, consequently resulting in myocardial injury^22^. Similar results were observed in our previous study, where we found *Rhodiola* increased weight-load swimming time and reduce cardiac injury after EE in normal mice, indicating that *Rhodiola* enhanced cardiac anti-stress capacity^5^. We previously demonstrated that oxidative stress remission and mitochondrial quality control adaptation, including fusion and fission, activated by *Rhodiola*, were the target mechanisms.

Clinical research has demonstrated that the exercise capacity of patients with IR and type 2 diabetes mellitus (T2DM) is limited^23^, while further evidence has established poor exercise capacity as an independent and strong predictor for all-cause mortality^24^. In this study, HFD mice presented decreased exercise capacity and worsened cardiac injury post-EE, indicating worse cardiac anti-stress capacity, which was alleviated following *Rhodiola* treatment. In skeletal muscle, another important target organ of IR, HFD mice have also been found to be worse affected than normal mice^25^. Noticeably, the inhibitor of NADPH oxidase, which is an important resource of ROS, relieved HFD-impaired mitochondrion function and exercise capacity. However, the role of disorder in the physiological antioxidant system is not fully known.

*Rhodiola* and its active constituent salidroside were found to be a natural antioxidant that protects mitochondria against injury in multiple diseases^18,26^. Nrf2, one of the targets of *Rhodiola*, is a key regulator of the cellular antioxidant response^19^. Besides oxidative stress, the activity of Nrf2 is also regulated by energy-based stimuli, thus, HFD inhibits Nrf2 and impairs the antioxidant system^27^. In this study, we found the expression of Nrf2 and downstream HO-1 were inhibited in HFD mice, while the antioxidant enzyme SOD2 and GPX4 were also inhibited, and the lipid peroxidation product MDA had increased. This implies the presence of a disorder of the antioxidant system in the myocardium of HFD mice which may contribute to the impaired cardiac anti-stress capacity. As expected, we found *Rhodiola* treatment redeemed the Nrf2-regulated antioxidant system and reduced oxidative stress. Future studies may focus their attention on structure optimization of salidroside to improve the targeting ability or pharmaceutical effect^28^.

In consideration of the coadjustment of oxidative and mitochondrial dynamics, we assessed the mitochondrial fusion and fission levels to further illustrate the mechanism behind *Rhodiola’s* protective effect. Fusion is a vital process preserving ATP production and ROS dissipation. Muscle from obese or T2DM patients show a reduced degree of fusion^29^. However, our results show that mitochondrial fusion marker MFN1, MFN2, and OPA1 increased in HFD mice after EE. Concerning the fission caused by damaged mitochondria, it was enhanced in response to HFD and EE stress in this study. We presume this discrepancy is a result of the difference in tissue type or pathological staging, as the fusion and fission level in this study was activated as physiological compensation for mitochondrial quality control and cardioprotection^30^. Futhermore, we found *Rhodiola* treatment, which improved ATP content in HFD mice myocardium, further facilitated mitochondrial fusion while inhibiting fission. This indicates that 8-week *Rhodiola* pre-intervened mice did not exhibit such severe injury in mitochondrial after EE or/and this injury was relieved by the self-protective quality control process including fusion. However, further studies with specific inhibitor administration or gene conditional knockout mice are needed to explore this issue.

**Figure 5:**
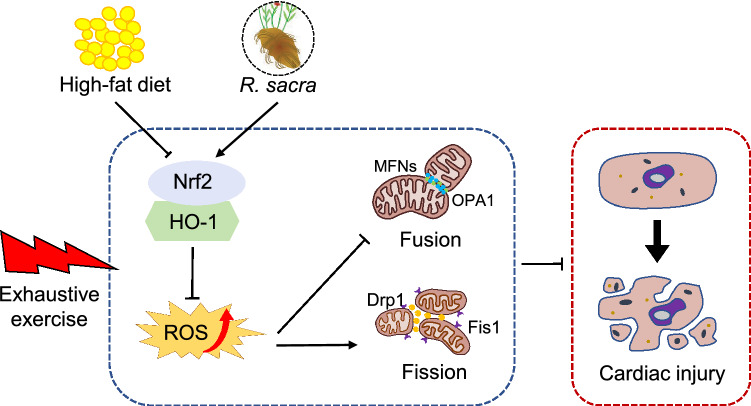
Proposed pathway of *Rhodiola* pre-conditioning enhances cardiac anti-stress capacity of Insulin Resistant Mice. Reactive oxygen species (ROS) production in cellular was closely associated with mitochondrial dynamics including fusion and fission. And mitochondrial dysfunction is critically involved in the acute stress-induced myocardial injury for mice. We found high fat diet (HFD) inhibited the antioxidant pathway Nrf2/HO-1 and aggravated the myocardial injury, while *Rhodiola* activated Nrf2/HO-1 pathway, reduced ROS production, regulated mitochondrial dynamics, and relieved the myocardial injury after exhaustive exercise.

In summary, this study used an EE mice model to simulate cardiac stress and explore *Rhodiola's* effect on the HFD-impaired cardiac anti-stress capacity in mice: *Rhodiola* activates Nrf2 signal and downstream antioxidant system and improves cardiac anti-stress capacity via regulating mitochondrial dynamic disorder, including fusion and fission (Fig. 5). The results conclude that Nrf2-mediated antioxidant enzyme and mitochondrial dynamic are the targets of *Rhodiola*, which may be a potential treatment to prevent cardiac event for T2DM and MS patients.

## Methods

### Ethics statement animal experimentation

8-week-old male C57BL/6J mice (20 ± 2 g) were bought from the Laboratory Animal Centre, Xiangya Medical School (Changsha, China). They were housed in temperature-controlled (22 °C ± 2 °C) quarters with a 12:12-h light–dark cycle and given free access to water and food. All animal procedures followed the guidelines for the use of live animals of the National Institute of Health and were approved by the Medicine Animal Welfare Committee of Xiangya Medical School, Central South University (Changsha, China). The initial approval ID (SYXK 2015-0017) was obtained in December 2015 and the re-approval ID (SYXK 2020-0019) was obtained in December 2020.

After 1-week adaptive feeding, mice were randomly divided into three groups (n = 16 for each): normal diet (ND), high-fat diet (HFD), and HFD + *Rhodiola* (HFD + RS). In HFD feeding 45% of total calories came from fat, while in ND feeding this figure is 18%. Mice were administrated with the same quantity of *Rhodiola* solution (5 mg/0.1 mL/10 g body weight) or normal saline (0.1 mL/10 g body weight) as a placebo by gavage every day for 8 consecutive weeks.

After 8 weeks, mice in each group were further divided into two subgroups: with or without an exhaustive exercise (EE) test (n = 8). Mice in the non-EE group were anesthetized via 1% pentobarbital sodium (150 mg/kg, i.p.) and then sacrificed via exsanguination after fasting, and collect blood samples from the inferior vena cava for lipid profile assays, visceral fat including mesenteric, epididymal, and perirenal fat tissue were dissected and weighted; Mice in EE subgroups underwent an exhaustive exercise as previously described^5^, while the researchers are blind to the groups. 12 h after the experiments, mice were anesthetized with 150 mg/kg pentobarbitone by intraperitoneal injection and then sacrificed, blood and myocardium samples were then collected. This study was conducted under the ARRIVE guidelines and any unnecessary animal suffering was avoided. The protocol has been described in Fig. 1A.

### Rhodiola

The extract from the root of *Rhodiola chrysanthemifolia subsp. sacra (Raym. -Hamet) H. Ohba (Rhodiola, RS)* was provided by the Tibet Rhodiola Pharmaceutical Holding Company. The extract complied with The Chinese Pharmacopeia 2015 (inspection report number C1051612067). The extraction method and the HPLC–MS analysis were described previously^31^. Briefly, the root of *R.sacra* was decocted twice for 1.5 h and 1 h respectively. The decoction was collected and dehydrated to powder after filtration. HPLC–MS analysis showed the proportion of main effective components, salidroside (C14H20O7, 2.62%) and flavone (C27H30O16, 3.27%). This powder was dissolved in distilled water at 50 mg/mL.

### Lipid profile assays

Serum was isolated from fasting blood by centrifugation at 4000 rpm for 10 min. The supernatants were collected and stored at -80 °C. Serum levels of total cholesterol, triglycerides, and low-density lipoprotein cholesterol were measured by the enzymatic method on an automatic biochemical analyzer (Beckman, IN, USA).

### HOMA-IR index

Blood glucose and insulin were respectively determined by glucometer (Accu-Chek Performa, Roche Diagnostics, Indianapolis, USA) and ELISA (CSB-E05071m, Wuhan, Hubei China). The HOMA-IR index was calculated as follows: HOMA-IR = fasting glucose (mmol/L) × fasting insulin (mU/L)/22.5.

### Exhaustive exercise test

After the 8-week intervention, forced weight-loaded swimming was performed as an exhaustive exercise (EE) test to assess cardiac anti-stress capacity^5,31^. Briefly, mice were forced to swim with a lead sheath of 5% of body weight till exhaustion, defined as the failure to rise above the surface of the water for 7 s.

### Exercise capacity

EE was performed in mice of the EE subgroup and the swimming time was recorded, which is regarded as exercise capacity as previously described^5^.

### Creatine kinase (CK), and blood urea nitrogen (BUN)

Blood samples were collected 12 h after the EE test. Serum was isolated from the blood sample by centrifugation. The CK concentration was determined by a colorimetric kit (Jiancheng, Nanjing, China), and the BUN was determined by a urease methods kit (Jiancheng), procedures are performed according to the manufacturer’s instructions.

### Transmission Electron Microscope (TEM)

Myocardium samples were collected after the EE test and resized to 1 × 1 × 3 mm^3^, then fixed and dehydrated. Next, we performed the embedding and solidifying of the tissue. After being sliced, tissues were observed by a transmission electron microscope (Tecnai G2 Spirit, FEI, United States).

### Western blot

Myocardium tissues were lysed in radioimmunoprecipitation assay (RIPA) buffer containing protease inhibitor (Beyotime). The protein concentrations of the lysates were measured by a BCA Protein Assay kit (Beyotime). After SDS-PAGE, the proteins were transferred to PVDF membranes with 0.45 μm pores (Millipore, MA, USA). After blocking with 5% non-fat dry milk for 2 h, the membranes were cropped to the kDa around the target proteins and then treated with primary antibodies against mitofusin1 (MFN1, 1:1000), MFN2 (1:2000), Dynamin-like 120 kDa protein (OPA1, 1:2000), dynamin-related protein 1 (DRP1, 1:1000) Mitochondrial fission 1 protein (FIS1, 1:1000), Nuclear factor erythroid 2-related factor 2 (Nrf2, 1:1000), Heme Oxygenase-1 (HO-1, 1:1000), Glutathione peroxidase 4 (GPX4, 1:1000), Superoxide dismutase 2 (SOD2, 1:4000), Glyceraldehyde 3-phosphate dehydrogenase (GAPDH, 1:3000), respectively. Following HRP-labeled goat anti-rabbit IgG or goat anti-mice IgG (1:5000, Proteintech), the membranes were incubated with ECL chemiluminescence solution (Thermo, Waltham, USA) and exposed to X-ray film for seconds to minutes. After developing and flushing, scan the film and analyzed the bands were analyzed using a gel documentation system (Bio-Rad, Hercules, CA, USA).

### Mitochondrial DNA ratio

The protocol was described previously^31^. Total DNA was isolated from myocardium tissue using a DNeasy Kit (Qiagen, GER). The succinate dehydrogenase complex subunit A (SDHA) and MtCO3 oligos were analyzed to evaluate the quantification of nuclear and mitochondrial genomes. The primers’ sequences are listed in Table 1.

**Table 1:** Primer sequences for qPCR analyses on tissues.

Gene	Primer	Product length (bp)
M-DNA-mt-Co3	GCAGGATTCTTCTGAGCGTTCT GTCAGCAGCCTCCTAGATCATGT	67
M-DNA-Sdha	TACTACAGCCCCAAGTCT TGGACCCATCTTCTATGC	194

### Adenosine triphosphate (ATP) content

The ATP content in the myocardium was determined via the phosphomolybdic acid colorimetric method (Jiancheng). Procedures were performed according to the manufacturer’s instructions.

### SOD2 and Malondialdehyde (MDA) assay

The xanthine oxidase method was used to determine the activity of SOD2 in myocardium tissues, and the thiobarbituric acid method was used to detect the content of MDA according to the manufacturer’s instructions respectively (Jiancheng).

### Statistical analysis

Statistical analyses were performed by Prism 6 software (GraphPad, Inc.).The results are expressed as Mean ± SD. One-way ANOVA plus the Student–Newman–Keuls test was used for statistical analysis. *P* < 0.05 represents statistical significance.

## Supplementary Information

Supplementary Information.

## Data Availability

The original contributions presented in the study are included in the article/ “Supplementary Material”, further inquiries can be directed to the corresponding author and S.L.
