# Supplementary material for: Rhodiola pre-conditioning reduces exhaustive exercise-induced myocardial injury of insulin resistant mice
Source: Sci Rep. 2022 Nov 23;12:20277. doi: 10.1038/s41598-022-20376-4 (PMC9700690; doi:10.1038/s41598-022-20376-4)
Supplement: Supplementary file 1 — Supplementary Information. [file 41598_2022_20376_MOESM1_ESM.pdf]

DATA IN BRIEF

Supplementary Figure 1

**fig1b.** swimming time (min)		
ND	HFD	HFD+RS
258.00	218.00	319.00
226.00	138.00	258.00
406.00	196.00	367.00
236.00	133.00	276.00
401.00	240.00	226.00
435.00	266.00	385.00

**fig1c.** CK after EE (kU/L)		
ND	HFD	HFD+RS
2.92	4.16	3.28
2.21	3.40	2.25
3.39	3.30	1.49
3.11	3.12	1.27
1.57	2.17	1.56
1.76	5.77	1.33

**fig1d.** BUN after EE (mmol/L)		
ND	HFD	HFD+RS
12.23	18.25	17.60
16.96	16.28	13.55
15.66	21.94	10.19
17.52	14.91	7.23
13.93	19.56	9.09
11.99	25.33	7.96

Supplementary Figure 2

**fig2a.** bodyweight before (g)		
ND	HFD	HFD+RS
19.71	22.60	22.58
20.17	23.08	21.26
25.89	20.03	22.28
19.58	20.57	25.49

22.39	23.00	25.65
21.77	22.41	24.64

bodyweight before (g)		
ND	HFD	HFD+RS
23.55	41.08	25.20
25.17	36.83	30.80
25.51	32.34	26.52
24.13	28.07	29.35
23.28	35.90	26.86
28.53	31.70	23.40

**fig2b.** adipose weight (g)		
ND	HFD	HFD+RS
1.01	1.82	1.13
1.08	2.81	2.53
1.33	3.49	2.20
1.79	3.69	1.87
0.95	3.45	1.83
1.43	2.76	1.82

**fig2c.** blood cholestrol (mmol/L)		
ND	HFD	HFD+RS
2.20	4.52	4.27
1.56	4.43	4.38
1.62	5.45	4.03
1.87	5.46	3.90
2.84	4.45	2.90
2.63	5.14	4.49

**fig2d.** blood triglyceride (mmol/L)		
ND	HFD	HFD+RS
0.65	0.89	0.85
0.78	0.92	0.81
0.72	0.87	0.70
0.88	0.96	0.82
0.74	0.92	0.81
0.58	0.99	0.79

**fig2e.** blood LDL-C (mmol/L)		
ND	HFD	HFD+RS
0.30	0.69	0.57
0.24	0.63	0.60

0.22	0.93	0.62
0.20	0.85	0.55
0.39	0.84	0.51
0.43	0.56	0.21

**fig2f.** blood glucose (mmol/L)		
ND	HFD	HFD+RS
8.30	9.20	8.70
7.20	9.80	9.20
8.40	9.50	9.20
7.70	9.60	7.50
7.80	9.70	8.50
8.80	9.60	7.50

**fig2g.** blood insulin (mIU/L)		
ND	HFD	HFD+RS
25.03	20.06	19.81
22.14	25.38	20.04
23.35	28.80	22.87
23.05	44.76	18.10
21.32	23.60	11.93
29.04	33.54	23.57

**fig2h.** HOMA-IR index		
ND	HFD	HFD+RS
9.23	8.20	7.66
7.08	11.06	8.19
8.72	12.16	9.35
7.89	19.10	6.03
7.39	10.17	4.51
11.36	14.31	7.86

Supplementary Figure 3

**fig3a.** SOD2 activity (U/mgprot)		
ND	HFD	HFD+RS
63.43	30.89	46.47
59.83	33.78	40.22
66.07	34.39	39.53

**fig3b.** MDA activity (nmol/mgprot)		
ND	HFD	HFD+RS
8.32	14.72	11.86

7.66	12.73	8.32
8.76	12.33	6.25

**fig3d.** NRF2 protein expression		
ND	HFD	HFD+RS
1.05	0.63	0.79
0.95	0.64	0.79
1.00	0.59	0.87
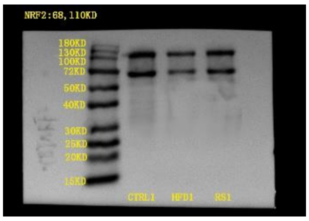	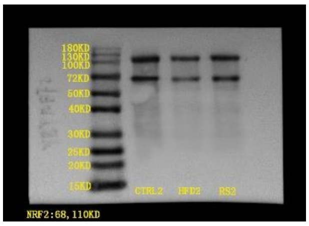	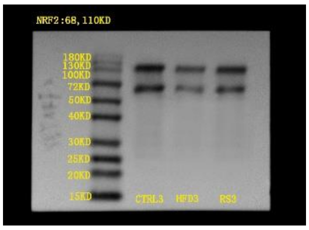
HO-1 protein expression		
ND	HFD	HFD+RS
0.90	0.17	0.47
1.00	0.40	0.57
1.10	0.37	0.60
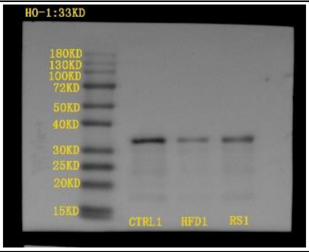	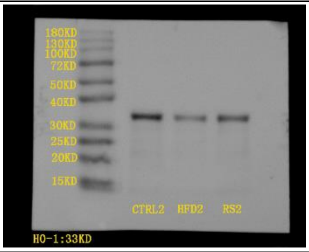	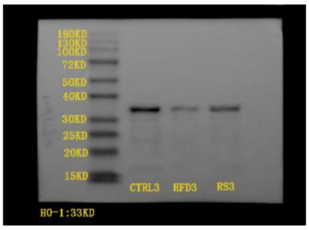
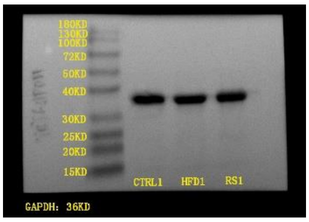	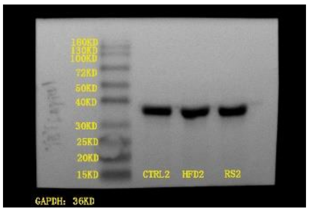	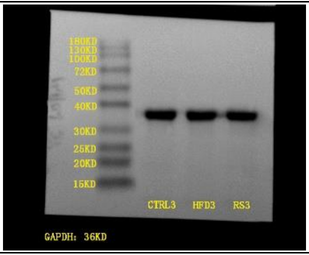

**fig3e.** GPX4 protein expression		
ND	HFD	HFD+RS
1.00	0.49	0.81
1.03	0.41	0.70
0.97	0.32	0.65
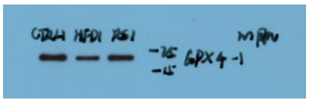	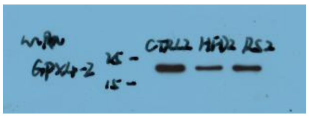	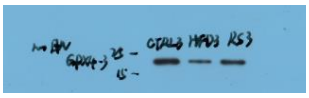
SOD2 protein expression		
ND	HFD	HFD+RS
0.93	0.48	0.74

1.06	0.45	0.72
1.01	0.29	0.77
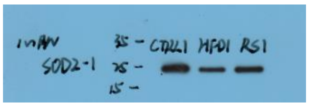	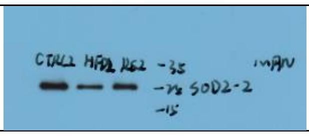	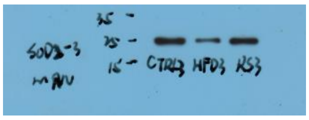
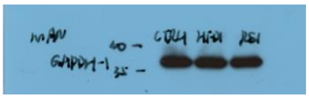	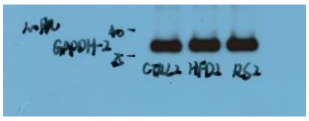	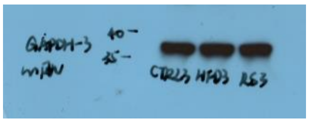

Supplementary Figure 4

**Fig4b.** MFN1 protein expression		
ND	HFD	HFD+RS
0.80	1.34	2.41
1.18	1.93	2.89
1.02	1.77	2.36
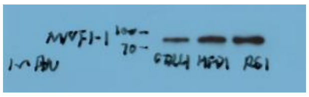	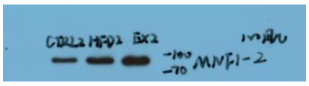	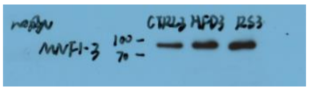
MFN2 protein expression		
ND	HFD	HFD+RS
1.22	2.92	3.57
0.81	2.03	3.00
0.97	2.35	3.24
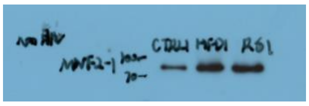	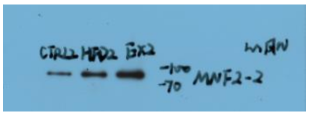	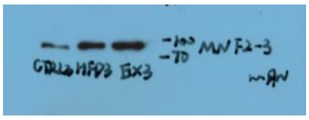
OPA1 protein expression		
ND	HFD	HFD+RS
0.88	3.38	5.50
1.50	3.63	4.38
0.63	2.88	5.13
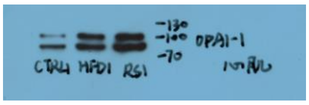	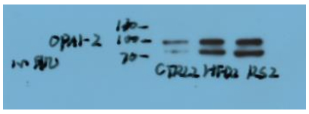	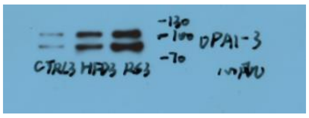
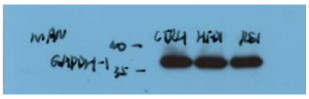	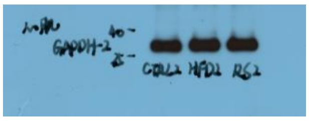	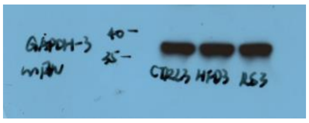

**Fig4c.** DRP1 protein expression		
ND	HFD	HFD+RS
1.00	2.50	2.07
1.21	3.79	3.07

0.79	2.86	2.36
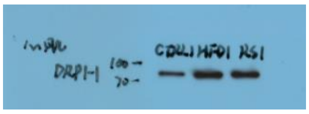	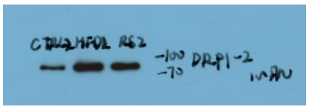	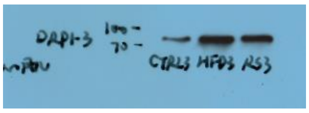
FIS1 protein expression		
ND	HFD	HFD+RS
0.83	3.17	2.08
1.00	3.33	2.67
1.17	3.33	2.42
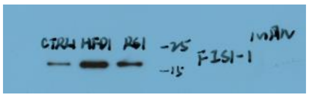	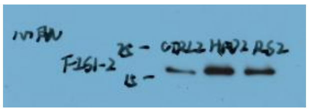	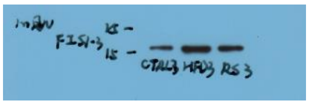
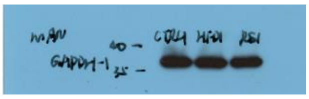	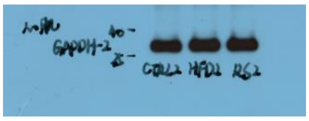	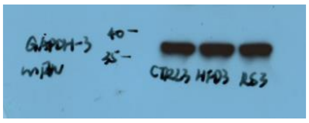

**Fig4d.** ATP content (umol/gprot)		
ND	HFD	HFD+RS
166.36	67.55	118.16
173.68	70.17	149.39
169.93	88.93	137.22
143.05	72.77	102.84
142.66	52.59	120.88
